# Application of a robotics path planning algorithm to assess the risk of mobile bearing dislocation in lateral unicompartmental knee replacement

**DOI:** 10.1038/s41598-022-05938-w

**Published:** 2022-02-08

**Authors:** Irene Yang, Jonathan D. Gammell, David W. Murray, Stephen J. Mellon

**Affiliations:** 1 Nuffield Department of Orthopaedics, Rheumatology and Musculoskeletal Sciences, University of Oxford, Oxford, UK; 2 Department of Engineering Science, Oxford Robotics Institute, University of Oxford, Oxford, UK

**Keywords:** Translational research, Biomedical engineering

## Abstract

Due to ligament laxity, bearing dislocation occurs in 1–6% of Oxford Domed Lateral (ODL) replacements with most dislocations occurring medially. Dislocations were studied using a previously built mechanical rig, however testing using the rig was inefficient. The aim of this study was to develop a better tool that was more reliable and efficient. An established robotics software package, the Open Motion Planning Library, was modified to accept the ODL components. Using a robotics path planning algorithm, the mobile bearing was allowed to find a way out from between the femoral and tibial components i.e. to dislocate. Testing assessed a range of clinically relevant positions of the femoral component relative to the tibial component. Dislocations were labelled as medial, lateral, anterior or posterior depending on the dislocation direction. The Distraction to Dislocation (DD) measured the minimum vertical distraction of the femoral component from the tibial component for a dislocation to occur. Results were validated against the mechanical rig. Statistical analysis of medial dislocation showed excellent agreement with an intraclass correlation value of 0.993 (95% CI 0.982–0.998). All DDs from the dislocation analysis tool were within 1 mm of the mechanical rig DDs with results sharing a remarkably similar trend. The robotics dislocation analysis tool output DDs which were marginally higher than the manual mechanical rig: 0.50 mm anteriorly, 0.25 mm posteriorly and 0.50 mm laterally. Medially, the computational DD differed on average by 0.09 mm (stand deviation: 0.2026 mm). Our study describes the development and validation of a novel robotics dislocation analysis tool, which allows mobile bearing dislocation risk quantification. The tool may also be used to improve surgical implantation parameters and to assess new implant designs that aim to reduce the medial dislocation risk to an acceptable level.

## Introduction

The Oxford Domed Lateral (ODL) unicompartmental knee replacement (UKR) implant was introduced to restore the normal anatomy and kinematics of the knee. Although the clinical results are good, 1–6% of mobile bearings dislocate^1,2^. Dislocations tend to occur in flexion as in this position, the lateral ligaments are lax, enabling lateral compartment distraction^3^. Dislocations theoretically are possible anteriorly, posteriorly, laterally or medially. Clinically, lateral dislocations do not occur, presumably because, when the knee is distracted allowing a dislocation, the lateral ligamants and soft tissues in the lateral knee prevent dislocations laterally. Anterior and posterior dislocation rates are acceptable and medial dislocations are the most common, with the bearing lodged at an angle above the tibial wall^2^. As such, this paper focused on the the most clinically relevant dislocation direction, medial dislocation.

The Open Motion Planning Library (OMPL) (Houston, Texas, USA) is an open-source path planning library consisting of various state-of-the-art sampling-based motion planning algorithms^4^. In robotics, path planning algorithms are frequently used to solve challenging problems encountered in autonomous motion. Path planning algorithms identify a series of steps connecting the robot’s start state to some goal state while avoiding collisions with the environment. Rapidly-exploring Random Trees (RRT)^5^ is a widely used path planning algorithm based on random sampling of robot states throughout the planning space.

This study reports the use of robotics path planning algorithm, RRT, and a modified OMPL Graphical User Interface (GUI), to construct a dislocation analysis tool to assess the minimum amount of vertical distraction of the femoral component relative to the tibial component that is required for a mobile bearing dislocation to occur.

## Methodology

**Figure 1:**
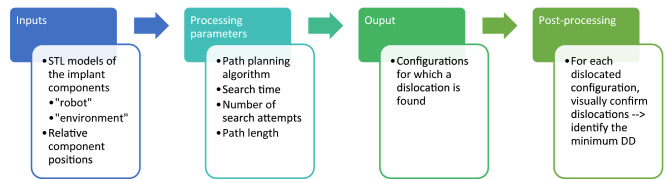
Flow chart to demonstrate how the dislocation analysis tool works.

In the dislocation analysis tool, the RRT algorithm is applied to the mobile bearing to allow the bearing to automatically identify a collision free path, should one exist, between the start (non-dislocated position) and the goal (dislocated position). If a collision free path is identified, the tool saves the configuration and these are later visually confirmed before identifying the DD for each mediolateral distance. The workflow is demonstrated in Fig. 1.

### The setup

Computer Aided Design (CAD) models of the ODL components (medium femur, C tibia, and size 3 bearing) were obtained from Zimmer Biomet (Swindon, UK). For the simulation, the OMPL GUI^4^ was modified to receive the ODL components: the femoral and tibial components defined the “environment” and the mobile bearing defined the “robot”.

For assessment, a computer with 64-bit Ubuntu 18.04.5 LTS operating system, x64-based processor an Intel Core i7-9850H CPU @ 2.60 GHz x 12 processor was used. The installed RAM was 16.0 GB (of which 15.4 GB was usable).

**Figure 2:**
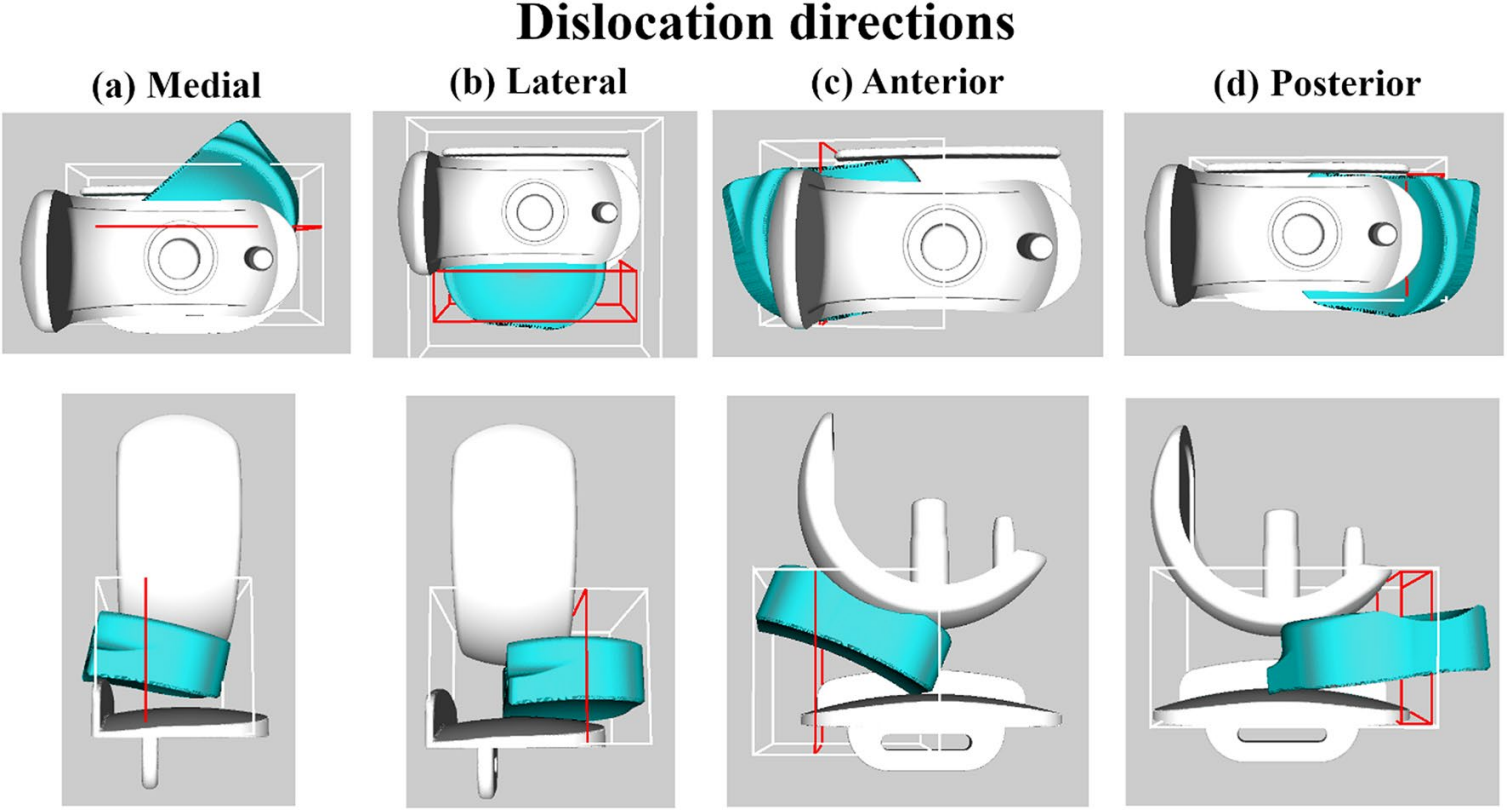
GUI setup showing mobile bearing dislocation with bounding boxes (white outline), goal area (red outline) for dislocation directions: (**a**) medial, (**b**) lateral, (**c**) anterior or (**d**) posterior dislocation. The top panel shows top view and the side view is shown in the bottom panel.

To assess dislocations, a bounding box was defined to limit the search area to the relevant region within the planning space (see Fig. 2).

**Figure 3:**
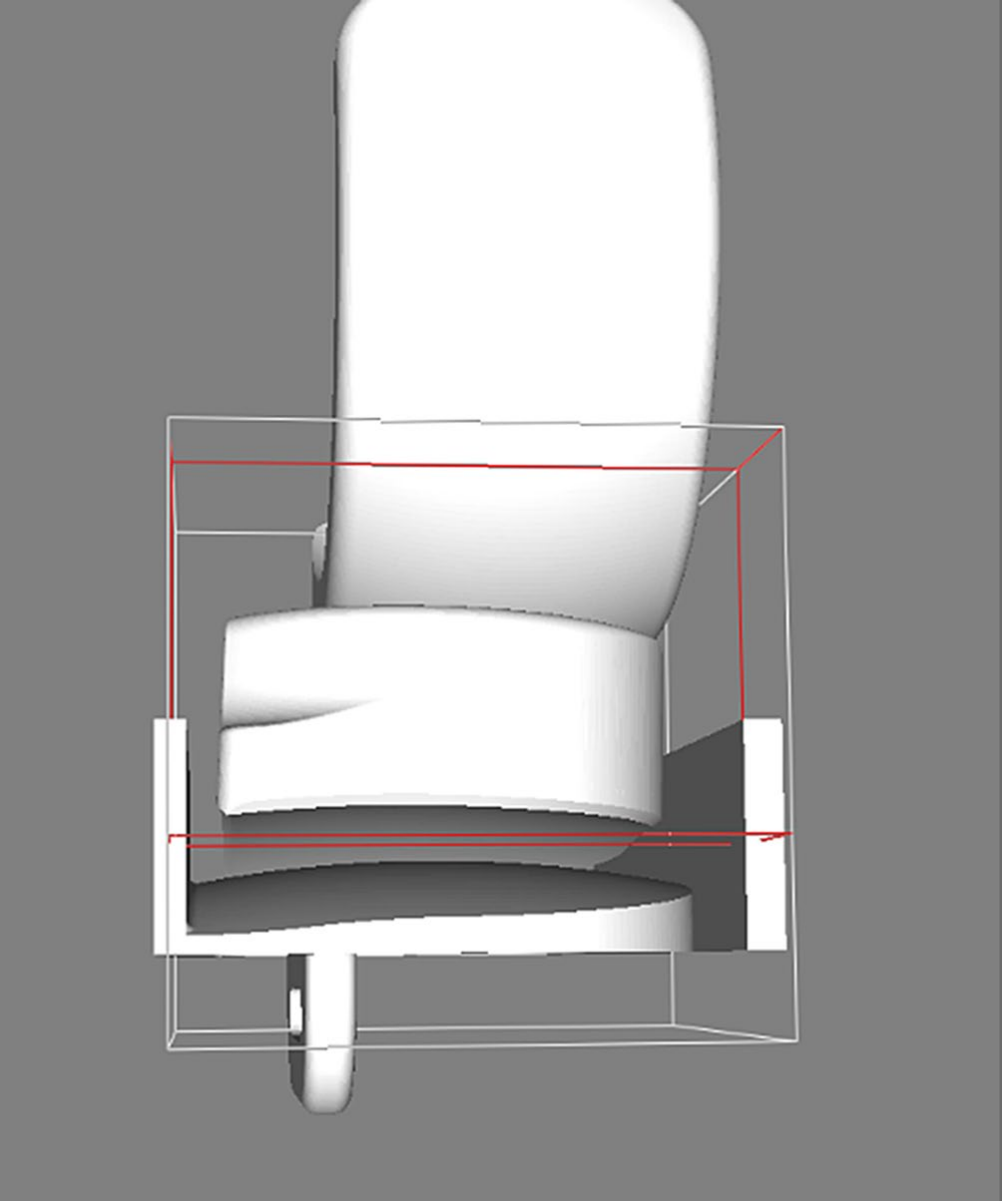
Additional wall added laterally for anterior/posterior dislocation testing.

Following intial testing where it was found that the bearing can dislocate anteriorly or posteriorly via the lateral space available, the bounding box for anterior and posterior dislocations was additionally supplemented with a lateral wall, to reproduce the lateral soft tissues that prevent the bearing from moving laterally (Fig. 3).

For medial, lateral, anterior and posterior dislocations, a “goal area” was defined which was used to bias the search direction and to identify dislocations. Dislocations occurred when the centre of mass of the mobile bearing entered the “goal area” (red outline in Fig. 2), located within the bounding box.

### Implementing the “smart search”

To improve the flexibility of the tool, the initial tool was modified to enable automatic identification of a valid start position which allowed the tool to correct for minor surface overlaps. This feature was particularly useful when, for some configurations, the bearing is translated mediolaterally causing minor overlap of the congruent surfaces between the inferior bearing surface and the domed tibial surface.

**Figure 4:**
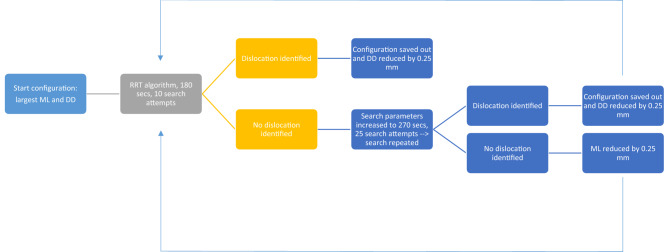
The coding loop used in the dislocation analysis tool for assessing dislocations.

The initial brute search algorithm was modified to enable smart searching (Fig. 4), whereby for each mediolateral distance, directional searching was implemented to allow the algorithm to begin the search from the configurations most likely to dislocate (maximum vertical distraction), to the configurations least likely to dislocate (minimum vertical distraction), starting with the smallest mediolateral translation distance. If no dislocations were identified in the allocated initial search time of 180 s and 10 search attempts, the configuration was re-searched with an extended time of 270 s and 25 search attempts. In other words, as the algorithm approached configurations that were least likely to dislocate (minimal vertical distraction between the femoral and tibial components), the search capacity was increased. If the extended search still failed to identify a dislocation, the algorithm would progress to assessing the next mediolateral (ML) translation distance. This was done to improve the likelihood of the tool to identifying more difficult dislocations whilst maintaining the efficiency of the search tool.

### Selecting search parameters

The RRT parameters (allowable search time & number of search attempts) were tested to select an improved testing time and number of searches. The relative distance between the femoral and tibial components was increased: vertically (0–8 mm, 0.25 mm increments) with mediolateral distance set at 2.00 mm (33 configurations). Based on a process of trial and error, the path step length was set to 1.25 mm as this value resulted in good performance for our tool and collision resolution was set to 0.0001 mm.

To determine the search time, the number of search attempts was fixed at 10 search attempts. The time for which the RRT algorithm was applied to the mobile bearing was increased from 15 to 405 s in 15 s increments (891 configurations) to assess whether dislocation was possible, and to record the minimum Distraction to Dislocation (DD).

To determine the number of searches, the RRT algorithm search time was fixed at 180 s. The number of attempts made to search for bearing dislocations was increased from 5 to 35 searches in 5 search increments to assess whether dislocation was possible, and to record the minimum DD.

### Testing

The starting position was defined with the bearing sitting flush against the tibial wall and congruent with the domed tibial surface. The relative distance between the femoral and tibial components was increased during testing and the testing parameters of DD and mediolateral translation were chosen based on physiologically relevant constraints. For the DD, as maximum distraction has been found to be 6.7 ± 1.9 mm^6^, testing assessed DD vertically from 0.00 mm to 8.00 mm in 0.25 mm increments. For ML translation, surgeons are unlikely to impant the components so the bearing is more than 6 mm from the wall, so testing assessed 0.00 mm to 6.00 mm in 0.25 mm increments. Therefore, a total of 825 configurations were tested.

For each configuration, the RRT algorithm was applied to the mobile bearing to assess whether dislocation was possible, and to determine the minimum DD. Path step length was set to 1.25 mm and collision resolution to 0.0001 mm. For each configuration, the RRT algorithm was allowed to run for 270 s and 10 searches. If no solution was found in this time, the search was repeated for the same configuration but with the time and search attempts extended to 405 s and 25 searches, respectively. Dislocations were labelled according to whether they occurred medially, laterally, anteriorly or posteriorly.

### Validation of dislocation results

**Figure 5:**
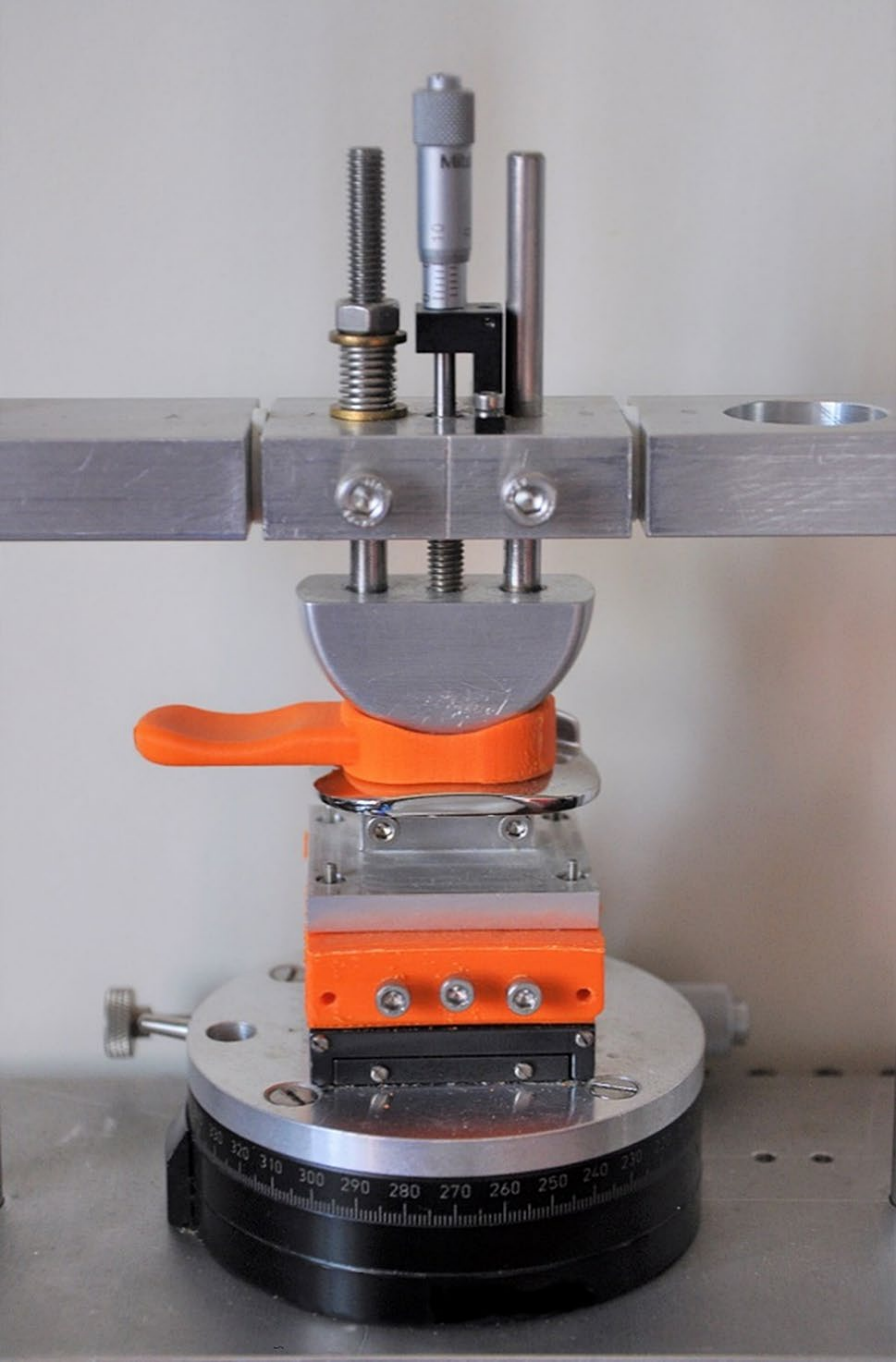
Mechanical rig used to study mobile bearing dislocation.

The final dislocation results were validated against data obtained using a previously described custom-built mechanical rig (Final design of the rig shown in Fig. 5)^7^. In the rig, the bearing was modified with a handle and 3D printed for testing. The modified bearing enabled manual manipulation of the mobile bearing into a dislocated position to aid in assessing dislocations either medially, anteriorly, posteriorly or laterally. To enable direct comparison, the same testing parameters as used in the robotics dislocation analysis tool were used in the mechanical rig: DD vertically ranged from 0.00 mm to 8.00 mm in 0.25 mm increments and mediolaterally from 0.00 to 6.00 mm in 0.25 mm increments, so a total of 825 configurations were tested.

### Medial dislocation statistical analysis

Since medial dislocation is the most clinically relevant dislocation direction, medial dislocation results were further analysed statistically. To compare the medial DD results obtained using the mechanical rig or the robotics tool, an Intraclass Correlation Coefficient (ICC) analysis was carried out: two-way random-effects model was used with type consistency and 95% confidence interval (CI) using IBM^®^ SPSS Statistical package (version 25) (SPSS Inc, Chicago, IL).

## Results

### Selecting optimal search parameters

**Figure 6:**
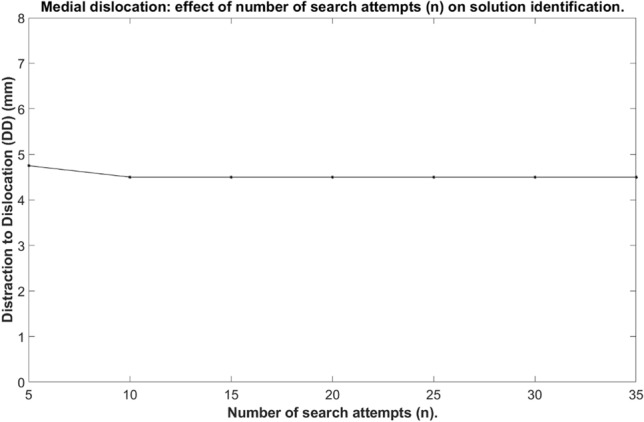
The effect of number of searches (n) on solution identification.

**Figure 7:**
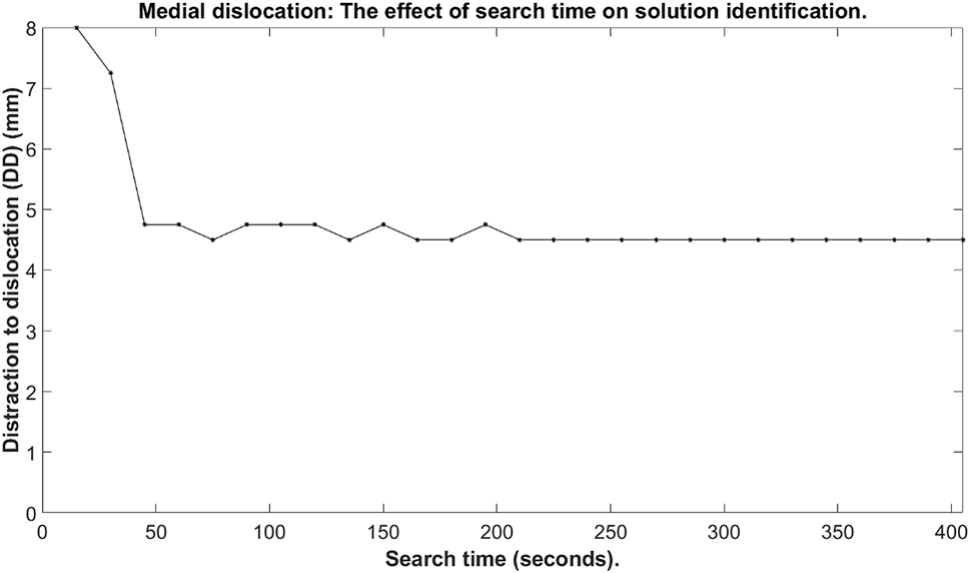
The effect of search time (s) on solution identification.

The OMPL GUI was successfully modified to receive the ODL implant components. Preliminary testing identified that at least 10 iterations (Fig. 6) and 210 s (Fig. 7) were required for maximum solution identification. However, within 10 search attempts (Fig. 6) and 45 s (Fig. 7) most of the solutions had been identified.

### Validation of dislocation results

**Figure 8:**
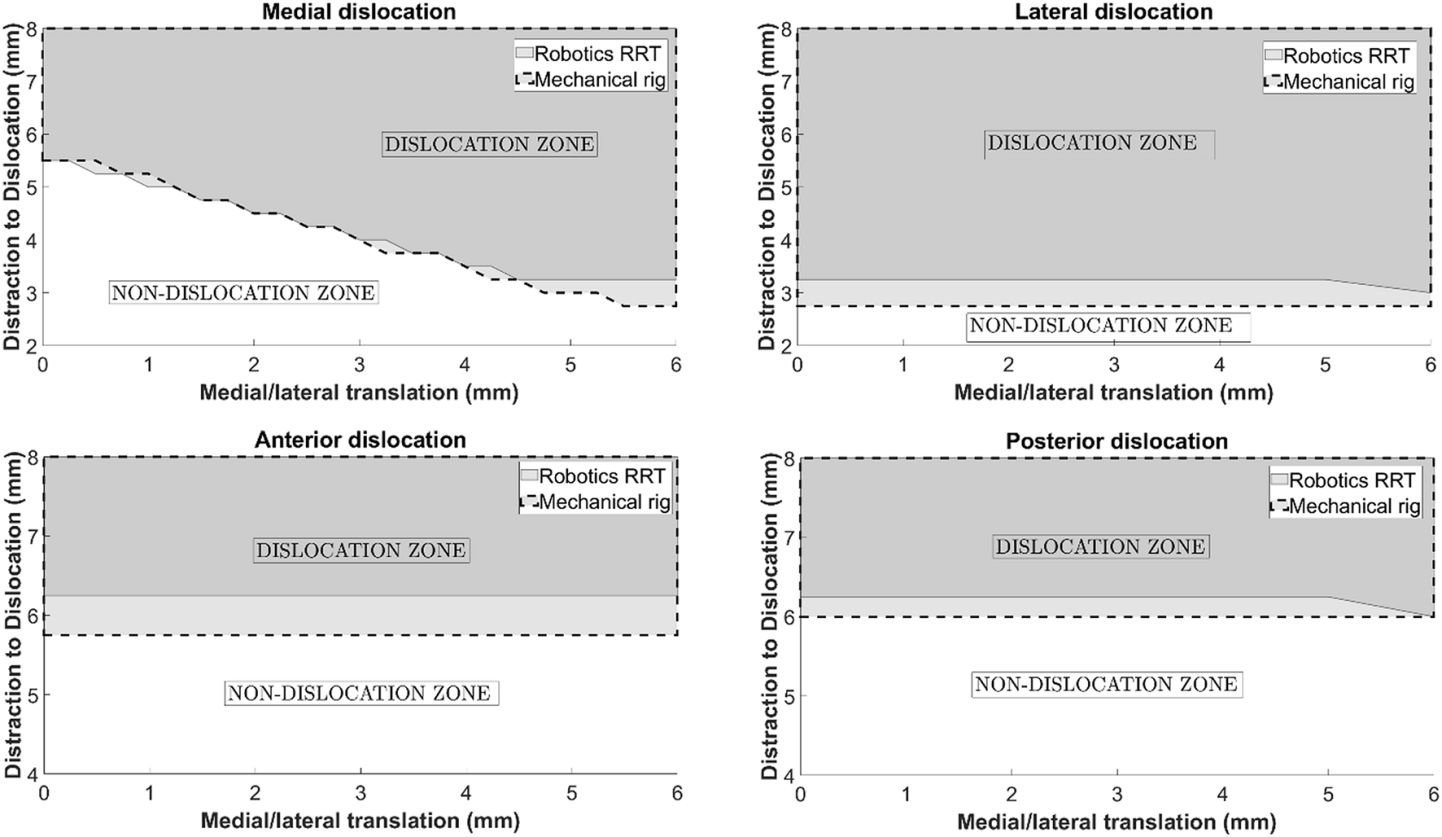
Mobile bearing dislocation (medially, laterally, anteriorly or posteriorly): the robotics dislocation analysis tool versus the mechanical rig. The grey region marks the dislocation zone while the white region marks the no dislocation zone.

The DD using the dislocation analysis tool was compared with that obtained using the mechanical rig for dislocations medially, laterally, anteriorly and posteriorly (Table 1 and Fig. 8). For both the mechanical rig and the dislocation analysis tool, anterior, posterior and lateral dislocations were independent of ML position, whereas medial dislocation was dependent on the ML position. For anterior, posterior and lateral dislocations, the robotics dislocation analysis tool output DDs which were marginally higher than the manual mechanical rig: 6.25 mm versus 5.75 mm anteriorly, 6.25 mm versus 6.00 mm posteriorly and 3.25 mm versus 2.75 mm laterally. For medial dislocation, as ML translation increases from 0.00 mm to 6.00 mm, the DD medially of the mobile bearing decreases. For the mechanical rig, the reduction was from 5.50 to 2.75 mm, and for the dislocation analysis tool, the reduction is from 5.50 to 3.25 mm (Table 1 and Fig. 8). The results appear to deviate between 4.50 and 6.00 mm of ML translation.

**Table 1:** Dislocation results obtained using the mechanical rig versus the robotics dislocation analysis tool.

Mediolateral translation (mm)	Distraction to Dislocation (DD) (mm)							
	Mechanical rig				Robotics dislocation analysis tool			
	Medial	Lateral	Anterior	Posterior	Medial	Lateral	Anterior	Posterior
0.00	5.50	2.75	5.75	6.00	5.50	3.50	6.25	6.25
6.00	2.75	2.75	5.75	6.00	3.25	3.00	6.25	6.00

### Medial dislocation statistical analysis

**Figure 9:**
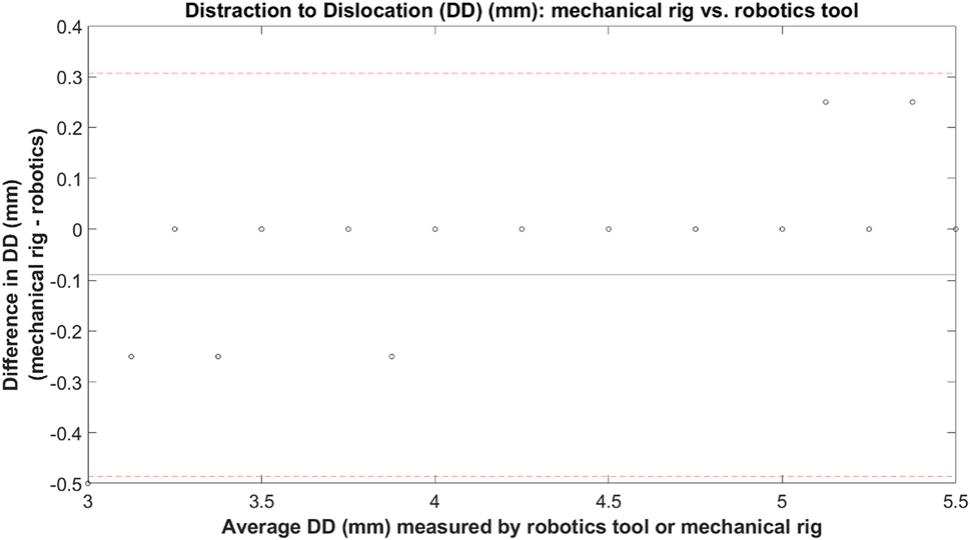
Bland–Altmann assessment for medial dislocation comparing mechanical rig versus robotics dislocation analysis tool.

Compared with mechanical rig results, the medial DD when using the dislocation analysis tool differed on average by 0.09 mm (standard deviation: 0.2026 mm) (Fig. 9).

When comparing medial dislocation results between the mechanical rig and the robotics analysis tool, the statistical analysis showed excellent reliability with an intraclass correlation value of 0.993 (95% CI: 0.982–0.998) based on a mean rating (k = 2), consistency type assessment and two-way random effects model.

## Discussion

Our study demonstrates the novel and successful application of a robotics path planning algorithm, RRT, to the mobile bearing, automating the movement of the mobile bearing from a non-dislocated position to a dislocated position to address the clinical problem of mobile bearing dislocation. We have demonstrated how doing so has resulted in the development of a dislocation analysis tool, which can be used experimentally to identify dislocations of the mobile bearing given various configurations of the femoral and tibial components. This enables us to study the effects of implant design and surgical technique on bearing dislocation.

The tool was optimised through implementation of directional searching (starting with the most likely dislocations to the least likely dislocations), with the ability to ramp up search parameters on approaching the configurations for which dislocation is less likely. Finally, the tool was also made more agile, by enabling automatic identification of a valid start state. Path planning algorithms like RRT are probabilistically complete in that the probability of finding a viable path (if one exists) from the start to the goal, is one if given infinite time and solve iterations to solve the problem^8^. However, in this study, not all environment configurations had a viable solution. Therefore, to ensure that all configurations that could result in dislocation were identified, it was decided to approximately double the search time and number of searches when encountering more difficult configurations where dislocations were less likely.

The dislocation analysis tool was made more efficient through the implementation of the directional searching feature and conditional ramping up of the search parameters. Directional searching meant that for each ML distance, once a confirmed non-dislocating configuration been encountered, the code would move to the next ML distance. The ramped up search parameters (increased search time and increased number of searches) were only activated once a non-dislocating configuration had been encountered. While, the exact testing time could not be calculated since RRT is based on randomly sampling points, the theoretical maximum time taken for testing could be calculated using the medial dislocation results: where 410 dislocations occured out of 825 configurations tested.

### Max theoretical testing time comparison

#### Non-optimised code

$$\begin{aligned} & {\text{Total}}\;{\text{configurations}}*{\text{search}}\;{\text{time}}*{\text{search}}\;{\text{attempts}} = {\text{total}}\;{\text{time}}\;{\text{for}}\;{\text{testing}} \\ & {825}\;{\text{configurations}}*{4}0{5}\;{\text{s}}*{25}\;{\text{search}}\;{\text{attempts}} = {8353125}\;{\text{s}}\;\left( {{96}.{67}\;{\text{days}}} \right) \\ \end{aligned}$$

#### Optimised code

$$\begin{aligned} & [({\text{Total}}\;{\text{dislocations}} - {\text{number}}\;{\text{of}}\;{\text{ML}}\;{\text{distances}}\;{\text{tested}})*{\text{search}}\;{\text{time}}*{\text{search}}\;{\text{attempts}}] \\ & \quad + [{\text{number}}\;{\text{of}}\;{\text{ML}}\;{\text{distances}}\;{\text{tested}}*{\text{extended}}\;{\text{search}}\;{\text{time}}*{\text{extended}}\;{\text{search}}\;{\text{attempts}}] \\ & \quad \quad = {\text{total}}\;{\text{time}}\;{\text{for}}\;{\text{testing}} \\ & [(410 - 25)*180*10] + [25*405*25] = 946125\;{\text{s}}\;\left( {{1}0.{95}\;{\text{days}}} \right) \\ \end{aligned}$$

While in practice the optimised code can take significantly less time to solve, especially for the configurations where there is more space for dislocation, implementing the changes to the code made the code almost nine times more efficient.

This study used the most common path planning alogorithm, RRT, for testing. Other path planning algorithms exist, which differ in how the problem is defined and how a solution is found^9^. Using a different planner may offer further improvements in the perfomance of the dislocation analysis tool (e.g. reduce search time and/or number of search attempts), however, for our purposes, optimising the RRT planner resulted in excellent results so it was not necessary to explore other planners.

The dislocation analysis tool was validated by comparing its results to the mechanical rig results. Using the robotics dislocation analysis tool, the DD medially differed by 0.09 mm (standard deviation of 0.2026 mm). However, with altering ML translation, the trends were the same suggesting the analysis tool is valid. The discrepancy between the results, particularly between 4.50 and 6.00 mm ML for medial dislocations, is likely due to experimental error with the mechanical rig. Using the rig, if enough force was applied, the bearing could dislocate, making dislocations with the rig “easier” than using the robotics tool. Further, under the applied force during testing, the 3D printed parts may have warped/been worn during testing which may have made dislocation with the rig easier than with the robotics tool, although this is not expected to be an issue as worn parts were replaced during testing. While in theory the discrepancy may also have originated from the robotics analysis tool, where inherent randomness of RRT may result in false negatives, we do not expect this to be an issue in our study since we ran a convergence test to optimise the search time and number of search attempts. Further refining the goal area may aid in identifying the more subtle dislocations where the bearing can lodge above the tibial wall, but does not completely come out from between the femoral and tibial components. Despite the minor discrepancy, results from the dislocation analysis tool showed excellent overall reliability when compared with the mechanical rig results with an ICC value of 0.993 (95% CI: 0.982–0.998). As such, the the dislocation analysis tool results were deemed sufficiently accurate.

As the laxity of the knee (and thus the maximal possible distraction in the knee) varies between patients, the risk of dislocation was assessed by the minimum amount of distraction that would allow a dislocation to occur given any setup of the femoral and tibial components. Results from the tool can be used to explain some aspect of the clinical problem of mobile bearing dislocation such as why medial dislocation is more likely to occur than anterior or posterior dislocation. The reason is because DD medially particularly at 6.00 mm ML (3.25 mm) was much less than that for an anterior or posterior dislocation (6.00 mm or 6.25 mm), though this significance reduces but remains less than DD anteriorly/posteriorly at 0.00 mm ML when the DD medially is 5.50 mm. Since surgeons are instructed to implant the components such that the bearing is as close as possible but not hitting the wall^7^, it is more likely that the ML is smaller rather than larger. While lateral dislocation required a similar amount of distraction to medial dislocation, particularly with ML > 4.50 mm, it was still less than the distraction required for a medial dislocation. However, more importantly, when the lateral compartment is distracted the lateral ligament and other soft tissues in the lateral knee are tight^3^, preventing a lateral dislocation.

In this study, for assessment of anterior/posterior dislocations, initial testing found that with the defined bounding box, the bearing was dislocating anteriorly/posteriorly via the lateral space available. To prevent this, a lateral wall was added. Addition of the lateral wall ensured that the dislocations were physiologically relevant anterior/posterior dislocations. Clinically, anterior and posterior bearing dislocation rarely occur and this dislocation rate is acceptable^2^. Tokuhara et al.^6^ studied the laxity of the lateral side of the knee in flexion and showed in an MRI study that the lateral compartment of the knee distracted on average 6.7 ± 1.9 mm following application of a varus stress to a knee joint in 90° of knee flexion. This is similar to the amount required for an anterior or posterior dislocation, probably explaining why these are rare. If the design of the implant could be modified so the amount of distraction for a medial dislocation was similar to that of an anterior or posterior dislocation, then dislocation risk would drop to an acceptable level.

While this study described the use of the tool using the ODL implant, the tool may be used in the same way with other mobile bearing UKR designs. Further, the tool has other uses beyond finding the DD. Firstly, the manner in which mobile bearings dislocate is not well understood. The tool has enabled us to gain insight for the first time, into the pathway that the bearing might be taking in order to reach a dislocated position. Further work to characterise this pathway, may enable us to gain a better understanding on the possible causes of dislocations. Secondly, since the tool can assess any setup of the femoral and tibial components, the tool may also be used to test the effects of surgical alignment on dislocation of the mobile bearing, or to identify the ideal parameters for surgical implantation of the ODL implant. Finally, the tool can be used to test new ODL implant designs, which may reduce the risk of mobile bearing dislocation.

The main limitation of the study is that it uses a computational model rather than being a cadaver or clinical study. There may be many factors which may cause the bearing to dislocate once the lateral compartment is distracted, but these are currently unknown. Some examples of possible factors are bow stringing of the popliteus tendon^10^, soft tissue impingement, buoyancy of the mobile bearing in the synovial fluid and anatomical structures which reduce the space available for the bearing to dislocate. However, none of these can be assessed using this computational model since like the mechanical rig, the robotics dislocation analysis tool is a static in vitro model which does not take into account the soft tissues surrounding the knee joint. However, in addition to replicating findings obtained using a mechanical rig in real life, the robotics dislocation analysis tool successfully provides an explanation for why medial dislocation is the most common mode of dislocation clinically. Further work on this tool would significantly increase the testing parameters, to ensure that search time and number of testing attempts, does not artificially eliminate possible dislocation configurations. Finally the goal area can be adjusted to ensure that the dislocations that are least likely are captured.

## Conclusion

Our study describes a novel and validated robotics dislocation analysis tool, which can identify mobile bearing dislocations (medially, laterally, anteriorly or posteriorly) given any setup or design of the femoral and tibial components of the ODL implant. Using the tool, the mobile bearing dislocation risk for any mobile bearing UKR can be quantified through the DD measurement. In addition to quantifying the dislocation risk, the tool may also be used to identify the ideal parameters for surgical implantation of the implant, or to assess potential new designs that increase the DD medially with the aim to reduce the risk of medial dislocations to an acceptable level.

## Abbreviations

ODL: Oxford Domed Lateral
DD: Distraction to Dislocation
UKR: Unicompartmental Knee Replacement
OMPL: Open Motion Planning Library
RRT: Rapidly-exploring Random Trees
GUI: Graphical User Interface
CAD: Computer Aided Design
ML: Mediolateral
ICC: Intraclass Correlation Coefficient
CI: Confidence Interval
MRI: Magnetic Resonance Imaging
